# Efficacy of sacubitril valsartan sodium tablet for the treatment of chronic heart failure: A systematic review protocol of randomized controlled trials

**DOI:** 10.1097/MD.0000000000018050

**Published:** 2019-11-22

**Authors:** Zhe Liu, Jing Wang, Yi Li

**Affiliations:** a Department of Cardiology, Yanan University Affiliated Hospital; b Department of Endocrine and Metabolism, Yanan University Affiliated Hospital; c Department of Geriatrics, Yan’an People's Hospital, Yan’an, China.

**Keywords:** sacubitril valsartan sodium tablet, chronic heart failure, randomized controlled trial, efficacy, safety

## Abstract

**Background:**

This study aims to systematically explore the efficacy of sacubitril valsartan sodium tablet (SVST) for the treatment of chronic heart failure (CHF).

**Methods:**

Nine electronic databases, including PUBMED, Cochrane Library, EMBASE, PsycINFO, Web of Science, Allied and Complementary Medicine Database, WANGFANG, Chinese Biomedical Literature Database, and China National Knowledge Infrastructure will be searched. Randomized controlled trials on SVST in the treatment of CHF will be collected. The search time limit will be from the establishment of each electronic database until June 1, 2019. Two authors will independently select the literature, carry out the data, and assess the methodological quality.

**Results:**

This study will systematically investigate the efficacy and safety of SVST for CHF. The outcomes consist of all-cause mortality, change in body weight, urine output, change in serum sodium; and incidence of any expected and unexpected adverse events.

**Conclusion:**

The findings of this study will summarize from evidence-based medicine and a scientific basis for the efficacy and safety of SVST in the clinical treatment of CHF.

**PROSPERO registration number:**

PROSPERO CRD42019138882.

## 1 Introduction

Chronic heart failure (CHF) is a complex progressive syndrome in which abnormal heart function results from structural and functional disturbances oxygen supply to heart tissues.^[1–4]^ It has been reported that CHF has become more and more common among the general population globally.^[5–8]^ It often causes high mortality, rate of hospitalization, very poor quality of life, and poor prognosis in patients with CHF.^[9–15]^ Therefore, to prevent its further progression and to treat such condition is very important for patients with CHF.

Sacubitril valsartan sodium tablet (SVST) has been reported to treat patients with CHF effectively.^[16–22]^ However, its results are still inconsistent. Thus, this study will systematically investigate the efficacy and safety of SVST for the treatment of patients with SVST.

## 2 Methods and analysis

### 2.1 PROSPERO registration

The protocol of this study has been registered on PROSPERO (CRD42019138882). Its report has followed the Preferred Reporting Items for Systematic Reviews and Meta-Analysis (PRISRMA) Protocol statement.^[23]^

### 2.2 Study inclusion and exclusion criteria

#### 2.2.1 Types of studies

All randomized controlled trials (RCTs) of the use of SVST in the treatment of CHF will be carried out with no limit to the language applied.

#### 2.2.2 Types of interventions

Experimental group: Patients in the experimental group has received SVST alone.

Control group: Patients in the control group has received any treatments, except any forms of SVST.

#### 2.2.3 Types of participants

Patients with CHF will be considered for inclusion regardless the race, sex, and age.

#### 2.2.4 Types of outcome measurements

The primary outcome consists of all-cause mortality. The secondary outcomes comprise of change in body weight, urine output, change in serum sodium; and incidence of all expected and unexpected adverse events.

### 2.3 Search methods for the identification of studies

#### 2.3.1 Electronic database searches

A total of 9 electronic databases include PUBMED, Cochrane Library, EMBASE, PsycINFO, Web of Science, Allied and Complementary Medicine Database, WANGFANG, Chinese Biomedical Literature Database, and China National Knowledge Infrastructure. All these databases have been searched from inceptions to the June 1, 2019 without language limitations. RCTs involving clinical efficacy and adverse events of SVST in the treatment of CHF will be collected. The retrieval strategy for PUBMED is presented in Table 1. Similar retrieval strategy for other databases will be built and utilized.

**Table 1:**
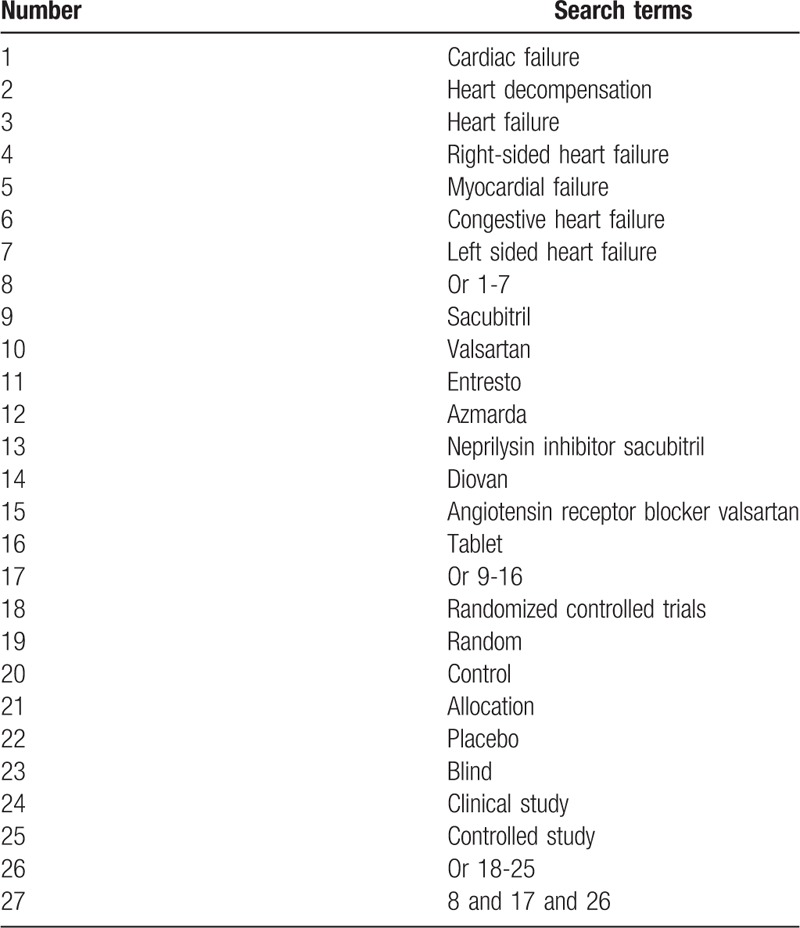
Search strategy of PUBMED.

#### 2.3.2 Other literature sources search

Other literature sources will also be searched, such as reference lists of relevant included RCTs and associated conference proceedings to avoid missing any potential studies.

### 2.4 Data collection and analysis

#### 2.4.1 Selection of studies

Two authors will independently screen and extract the electronic literature according to the pre-designed eligibility criteria. A third author will be invited to solve any divergences between 2 authors. All titles and abstracts of all records will be scanned to exclude any irrelevant records initially. After that, all remaining records will be read by full texts to judge whether the literature meet all eligibility criteria. The study selection process will be presented in the PRISRMA flowchart.

#### 2.4.2 Data extraction and management

The following data will be extracted by two authors independently. Any divergences between 2 authors will be resolved by a third author through discussion. The information comprise of basic information (title, author, publication time, etc), types and methods of trials (sample size, randomization, concealment, blinding, etc), patient characteristics (gender, age, inclusion, and exclusion criteria, etc), treatment details (type of intervention, dose, mode, treatment course, etc), and outcome indicators (all outcomes, adverse events, follow-up results, etc).

#### 2.4.3 Missing data dealing with

If there is any insufficient or missing data, we will contact original authors using email. If these data are not available, we will only analyze the available data, and will also explore its possible impacts.

### 2.5 Study quality assessment

Two authors will independently judge the study quality using Cochrane risk of bias tool. When there are disputes regarding the assessments, issues raised will be solved after being fully discussed by a third author involved. It includes 7 parameters in accordance with the standard guidelines of RCTs assessment manual of Cochrane Library. Each parameter will be further assessed by low, unclear, or high risk of bias.

### 2.6 Statistical analysis

We will apply RevMan 5.3 software to perform statistical analysis. For continuous variables, mean difference or standard mean difference and 95% confidence intervals will be expressed. For dichotomous values, risk ratio and 95% confidence intervals will be expressed.

*I*^*2*^ test is used to check heterogeneity among eligible studies. *I*^*2*^ ≤ 50% means having minor heterogeneity, a fixed-effects model will be used to pool the data, and meta-analysis will be carried out. On the other hand, *I*^*2*^ > 50% means having substantial heterogeneity, a random-effects model will be utilized for data synthesizing, and subgroup analysis will be conducted. If there is still significant heterogeneity after subgroup analysis, meta-analysis will not be carried out. Instead, a narrative summary will be described.

### 2.7 Additional analysis

#### 2.7.1 Subgroup analysis

We will perform subgroup analysis to explore any possible reasons of substantial heterogeneity according to the different characteristics, treatments and compactors, and outcomes.

#### 2.7.2 Sensitivity analysis

We will also carry out sensitivity analysis to investigate the robustness and stability of combined results by removing studies with high risk of bias.

#### 2.7.3 Reporting bias

We will check any reporting bias by using funnel plot and Egger regression test when more than 10 eligible RCTs are included.^[24,25]^

### 2.8 Ethics and dissemination

Ethical approval is not needed in this study, because it will not analyze individual data. The findings of this study are expected will be disseminated at peer-reviewed journals.

## 3 Discussion

Previous studies have hypothesized that SVST plays a very important role in the treatment of patients with CHF. However, its results are still inconsistent, and all evidence is still at the conceptual level. Thus, we will systematically assess the efficacy and safety of SVST for the treatment of CHF. This study will summarize the most recent evidence of SVST for CHF. The results of this study may provide very helpful evidence for the clinicians, future researches, and health related policy maker.

## Author contributions

**Conceptualization:** Zhe Liu, Jing Wang, Yi Li.

**Data curation:** Zhe Liu, Jing Wang, Yi Li.

**Formal analysis:** Zhe Liu, Jing Wang, Yi Li.

**Investigation:** Yi Li.

**Methodology:** Zhe Liu, Jing Wang.

**Project administration:** Yi Li.

**Resources:** Zhe Liu, Jing Wang.

**Software:** Zhe Liu, Jing Wang.

**Supervision:** Yi Li.

**Validation:** Zhe Liu, Jing Wang, Yi Li.

**Visualization:** Zhe Liu, Jing Wang, Yi Li.

**Writing – original draft:** Zhe Liu, Jing Wang, Yi Li.

**Writing – review & editing:** Zhe Liu, Jing Wang, Yi Li.
